# Non-invasive imaging in heart transplant recipients: state of art and future direction

**DOI:** 10.1007/s00259-026-08045-4

**Published:** 2026-07-14

**Authors:** Teresa Mannarino, Roberta Assante, Emilia Zampella, Adriana D’Antonio, Erica Di Donna, Marianna Crusco, Alberto Cuocolo, Wanda Acampa

**Affiliations:** Department of Advanced Biomedical Science, University Federico II of Naples, Via Pansini 5, Naples, 80131 Italy

**Keywords:** heart transplantation-CAV-noninvasive imaging-precision medicine

## Abstract

Imaging in heart transplantation is rapidly evolving from a primarily descriptive tool to a central component of predictive, precision-based graft surveillance. This review summarizes current and emerging roles of non-invasive imaging across the continuum of post-transplant care. Starting from the traditional use of imaging modalities such as echocardiography, computed tomography, magnetic resonance, nuclear imaging techniques, the focus is centered on the most recent evidence on the established techniques and the more recent advances, delineating gaps, and future directions towards routine clinical implementation. Overall, the review supports a paradigm shift in which multimodal, quantitative, and AI-enabled “precision imaging” could become integral to tailoring surveillance strategies, guiding therapy, and optimizing long-term outcomes for heart transplant recipients.

## Introduction

Heart transplantation (HTx) is the preferred treatment for selected patients with advanced heart failure, offering a valid life-extending option when optimal medical therapy fails. Thanks to advances in surgical techniques, immunosuppressive therapy and clinical monitoring, the median survival rate now exceeds 13 years [1]. However, this increased longevity has also led to a rise in chronic complications, such as acute and chronic rejection, cardiac allograft vasculopathy (CAV), infection and fibrosis. Traditionally, post-transplant surveillance has relied on endomyocardial biopsy (EMB) and invasive coronary angiography (ICA) which are considered the respective reference standards for the detection of acute cellular/antibody-mediated rejection and CAV [2]. Although these techniques remain central in contemporary clinical practice, they are burdened by some limitations. EMB, still considered as the gold standard for rejection grading, is invasive and associated with procedural risks such as ventricular perforation, tamponade, arrhythmias, valve damage, and infection [2]. Moreover, its diagnostic accuracy is constrained by sampling error and interobserver variability, given the focal and heterogeneous nature of rejection, which may lead to underestimation or delay in diagnosis [3]. Similarly, ICA provides an excellent luminal assessment but lacks sensitivity for detecting early, diffuse intimal thickening and microvascular dysfunction which represent pathophysiological markers of early-stage CAV [4, 5]. These subtle forms of disease often precede overt angiographic stenoses and are better characterized by intravascular ultrasound (IVUS), optical coherence tomography (OCT), or advanced physiological testing such as coronary flow reserve (CFR) and microcirculatory resistance indices [6, 7]. Therefore, the reliance on EMB and ICA alone cannot be considered as an anticipatory surveillance algorithm, potentially missing the window for early intervention before irreversible allograft injury arises [8]. Over the past decade, non-invasive cardiac imaging has undergone a paradigm shift from qualitative assessment toward quantitative, multiparametric, and molecular approaches, enabling deeper phenotyping of different cardiac pathology. Thus, despite echocardiography remains the first-line tool for routine functional assessment, advanced modalities such as computed tomography (CT), cardiac magnetic resonance (CMR), and positron emission tomography (PET) now provide complementary insights into myocardial structure, tissue composition, perfusion, and inflammation [5], prior to phenotypic manifestation of graft failure, offering the potential to predict which patients are at higher risk of adverse outcomes [9]. In addition, by integrating multiparametric measures such as tissue mapping, perfusion quantification and myocardial flow reserve (MFR) from the different imaging modalities with emerging computational analytics of radiomics and machine learning, we can move towards individualized risk stratification and early therapeutic intervention [9]. It has been considered that echocardiography and endomyocardial biopsy are routinely performed according to time-defined follow-up protocols recommended by current International Society for Heart and Lung Transplantation (ISHLT) guidelines [10]. Otherwise, the optimal timing, frequency, and clinical positioning of advanced non-invasive modalities such as CMR, PET, and coronary CT angiography remain less clearly established. These advanced imaging techniques are currently used heterogeneously across centers evaluating different endpoints such as risk stratification, response to clinical deterioration or abnormal biomarkers, or serial imaging evaluations in selected high-risk patients, due to a variability in local expertise and resource availability. The aim of the present review is to overview the most recent findings and to critically evaluate the evolving role of non-invasive imaging in heart transplant surveillance, highlighting the transition from descriptive assessments to predictive, multimodal, and quantitative approaches capable of early identification of HTx complications for a possible personalizing long-term patients’ management.

## Traditional role of imaging and emerging quantitative imaging biomarkers

Imaging has long played a pivotal role in graft surveillance, assessing function, and identifying vascular complications in patients with HTx. Imaging techniques may raise suspicion for rejection and identify indirect functional or structural abnormalities contributing to clinical decision-making. Transthoracic echocardiography (TTE) and ICA represent traditional approaches for the evaluation of HT patients. TTE remains the first line tool for routine functional assessment of the transplanted heart because of its safety, availability, and ability to provide serial bedside monitoring of ventricular volumes, systolic/diastolic function, valvular integrity, systolic pulmonary artery pressure and pericardial status [11]. In more recent times, advanced echocardiographic techniques such as tissue Doppler imaging and speckle-tracking strain have been shown to detect subtle myocardial dysfunction associated with acute rejection and early graft vasculopathy [12]. Specifically, global longitudinal strain in both left and right ventricles are known to be significant predictors of mortality in HTx patients with an episode of acute rejection [13]. Despite this, echocardiography is limited by comparatively low sensitivity for microvascular disease or early inflammatory injury changes in global ejection fraction, whereas conventional Doppler parameters may occur only late in the disease process [14]. For coronary surveillance of CAV, ICA has been the guideline-recommended standard. However, angiography assesses luminal stenoses but lacks sensitivity for diffuse intimal thickening, concentric remodeling, or microvascular involvement [6]. To enhance sensitivity, intravascular ultrasound (IVUS) has been used as a complementary tool: previous studies demonstrated that intimal thickening and vessel wall changes detected by IVUS within the first post-transplant year strongly predicted late CAV and adverse outcomes [15]. Thus, while ICA/IVUS remain central for epicardial coronary assessment, they are invasive and do not fully capture microvascular or perfusion abnormalities. In the evolving landscape of graft surveillance after HTx, the shift toward quantitative and multiparametric imaging biomarkers offers considerable promise for early detection and prognostication of graft injury and vasculopathy (Tables 1 and 2).

**Table 1:** Overview of the main non radionuclide diagnostic imaging techniques used for HTx patients’ assessment

Imaging Modality	Primary Target / Biomarker	Advantages	Limitations
Echocardiography	Ventricular volumes, systolic and diastolic function, valvular status	Widely available, low cost, real-time monitoring, sensitive to early dysfunction	Low specificity for rejection; low sensitivity for microvascular disease; limited tissue characterization
Computed tomography	Calcium scoring, coronary luminal assessment, plaque burden, epicardial coronary anatomy and related derived functional parameters	Non-invasive alternative to invasive angiography	Limited spatial resolution
Cardiac MR	Ventricular volumes and function, LGE for focal fibrosis, edema	Excellent tissue characterization, non-ionizing, rejection-related inflammation	Cannot detect early diffuse fibrosis; contrast use; lack of standardized cut-offs across centers

**Table 2:** Overview of the main radionuclide diagnostic imaging techniques used for HTx patients’ assessment

Imaging Modality	Tracers	Primary Target	Imaging Parameters	Advantages	Limitations
Cardiac SPECT [34, 35]	[^99m^Tc]Tc-sestamibi [^99m^Tc]Tc-tetrofosmin	Perfusion Function	SSS, SRS, SDS LVEF, EDV, ESV, WMS	Non-invasive Widely available Combined perfusion and functional assessment Useful for follow-up and risk stratification	Limited sensitivity for early diffuse CAV Low spatial resolution Attenuation artifacts Limited quantitative assessment
Cardiac PET **[** 39 **–** 42 **]**	[^15^O]H_2_O [^13^N]NH_3_ [^82^Rb]Rb	Perfusion Function Coronary blood flows and microvascular integrity	SSS, SRS, SDS LVEF Baseline MBF, Hyperemic MBF MFR	Quantitative Reproducible Early detection of CAV Comprehensive single examination	Limited availability High cost
	[^18^F]FDG	Inflammation and rejection activity	SUV, TLG, CMV	Sensitive to immune cell activation	Patient preparation Lack of data on HTx
	[^68^Ga]FAPI	Fibroblast and macrophage activation	Target/background ratio	Specific molecular insight into fibrotic and inflammatory pathways	Mostly experimental No data on HTx Approval pending

*SSS*, summed stress score; *SRS*, summed rest score; *SDS*, summed difference score; *LVEF*, left ventricular ejection fraction; *EDV*, end diastolic volume; *ESV*, end systolic volume; *WMS*, wall motion score; *MBF*, myocardial blood flow; *MFR*, myocardial flow reserve; *SUV*, standardized uptake value; *TLG*, total lesion glycolysis; *CMV*, cardiac metabolic volume; *CAV*, cardiac allograft vasculopathy; *HTx*, heart transplanted

### Quantitative approach by cardiac computed tomography

The traditional role of CT in HTx surveillance was dedicated mainly on post-operative and structural complications, then for the graft functional testing. Some authors in late 1990s and 2000s reported the use of CT as a helpful tool to evaluate mediastinal hematomas, pericardial collections, pseudoaneurysms, pulmonary artery or vein complications, and extracardiac thoracic pathology when chest radiography or echocardiography were inconclusive [16]. Electron-beam CT (EBCT) and first-generation multidetector CT were investigated for calcium scoring and coronary lumen assessment in CAV but limited spatial and temporal resolution led to suboptimal sensitivity and specificity compared with ICA and IVUS [17]. With technological advancement, emerging quantitative metrics by coronary CT angiography (CCTA) are gaining traction in transplant surveillance. Published reviews in 2025 indicate that CCTA shows promising sensitivity (84.8%; 95% Crl 88.8%-89.4%) and specificity (99%; 95% Crl 98.4%-99.4%) for detection of significant CAV at segment level, and new findings suggest that CT-derived plaque burden may correlate with adverse graft outcomes [18]. Further, CT may enable assessment of diffuse, concentric intimal thickening not appreciated on conventional angiography, thereby enhancing early phenotyping of CAV. In a previous study Nous et al. [19] have demonstrated by using CCTA as the primary surveillance tool after HTx, that it was feasible in 75% of the cohort, with good to excellent image quality in 92%, low radiation dose and identification of obstructive CAV in 15%, while only a small minority required downstream invasive angiography or revascularization, underscoring its suitability as a first-line screening test. Following on this, Budde et al. [20] reported the first systematic use of fractional flow reserve (FFR) by CCTA for functional coronary artery evaluation in the follow-up of 73 HTx patients, demonstrating the presence of diffuse physiological impairment that is not apparent on ICA alone. Sharma et al. [21] extended this concept longitudinally, showing that over a 2-year interval serial CCTA with FFR revealed a modest but consistent decline in distal FFR and an increase in focal stenoses, suggesting that temporal FFR changes may serve as a quantitative marker of CAV progression and a trigger for intensifying medical therapy. Beyond physiology, Ahn et al. [22] combined CCTA with dynamic CT myocardial perfusion imaging demonstrated that the combination of stenosis severity by CCTA and minimum myocardial blood flow (MBF) yielded excellent diagnostic performance (AUC 0.89, CI 0.780–0.952) for invasive CAV defined by ICA+IVUS, supporting an anatomical-functional CT protocol within a single examination [22]. Furthermore, Wall et al. [23] introduced inflammatory and remodeling metrics derived from CCTA, showing that pericoronary adipose tissue (PCAT) density was higher in patients with CAV and that PCAT density and vessel volume-to-myocardial-mass-ratio (V/M) significantly improved CAV prediction (AUC from 0.75 to 0.85 when added to donor features); a PCAT density greater than − 66 HU was strongly associated with all-cause mortality and a composite endpoint of death, CAV progression, acute rejection and coronary revascularization over 5 years [23].

It should be noted that FFR-CT and CT-derived MBF assess different aspects of coronary pathophysiology. FFR-CT is a computational fluid dynamics-based estimate of the pressure drop along a coronary artery and does not directly measure myocardial blood flow, whereas CT-MBF is instead a dynamic contrast-enhanced acquisition that estimates absolute myocardial perfusion through pharmacokinetic modelling, and is therefore more directly comparable to PET-derived MBF. This distinction is relevant because the two parameters reflect different pathophysiological domains (epicardial hemodynamic significance versus microvascular perfusion) and have different implications for CAV characterization.

### Quantitative approach by cardiac magnetic resonance

For myocardial structural assessment beyond function, cardiac magnetic resonance (CMR) has increasingly been applied in heart transplant recipients. Traditionally, CMR has played a complementary role in HTx surveillance. Early work focused on using T2-weighted imaging and later T2 mapping to detect myocardial edema as a non-invasive marker of acute rejection has shown that prolonged T2 relaxation times correlate with biopsy-proven rejection episodes [24, 25]. CMR provides detailed tissue characterization of fibrosis, edema, and microstructural injury [24]. In parallel, late gadolinium enhancement (LGE) by CMR was adopted to identify focal myocardial fibrosis and scar, with studies in adult and pediatric heart-transplant recipients demonstrating that LGE is relatively common and associated with prior rejection burden and adverse remodeling [26, 27]. CMR has shown promise in identifying graft injury and stratifying risk, but its application in large transplant cohorts remains limited and it can be affected by contraindications, such as renal impairment or implanted devices [8]. In particular, the presence of cardiac implantable electronic devices or retained/abandoned leads from pre-transplant devices may represent a limitation. Complete lead extraction at the time of transplantation is not always feasible because of procedural complexity and associated risks. Consequently, retained leads may limit the use of CMR because of safety concerns, institutional restrictions, or significant susceptibility artefacts, potentially reducing image quality and diagnostic accuracy.

Advanced CMR mapping techniques, including native T1, T2 mapping, extracellular volume (ECV) quantification, and LGE have emerged as powerful tools to detect subclinical rejection and diffuse myocardial fibrosis. The diagnostic performance of the different CMR imaging parameters in the evaluation of acute rejection was evaluated in a metanalysis [28]. Using EMB as the reference standard it was demonstrated that T2 mapping showed the best overall performance, with an AUC = 0.92 (95% CI: 0.89–0.94), significantly higher than the other parameters. Native T1 mapping showed a good performance with an AUC = 0.84 (95% CI: 0.81–0.87), while ECV demonstrated very high sensitivity (91.3%) but more modest specificity (67.6%), consistent with ECV being a sensitive but less specific marker of rejection, likely influenced by chronic fibrosis and vasculopathy [28]. In contrast, binary LGE (presence/absence) had poor diagnostic accuracy for acute rejection (AUC = 0.56, 95% CI: 0.51–0.60), although the authors emphasize that LGE burden is prognostically important for long-term outcomes in transplant recipients [28]. Overall, the meta-analysis supports a central role for quantitative CMR mapping as non-invasive markers of myocardial edema and interstitial expansion in acute rejection and proposes multiparametric CMR as a promising gatekeeper to reduce the need for routine EMB in heart-transplant surveillance. More recently, it has been demonstrated that, in a pediatric/adult cohort, global T1 and T2 values elevated during acute rejection were associated with donor-derived cell-free DNA levels (an emerging biomarker of graft injury) [29]. Moreover, published findings indicate that CMR mapping metrics (T1 ≥ 1061 ms, T2 ≥ 50 ms and ECV ≥ 31.4%) not only detect microstructural injuries such as edema or diffuse fibrosis, but also stratify risk of adverse outcomes, supporting their role as prognostic biomarkers rather than purely descriptive tools [30]. Quantitative CMR tissue parameters clearly differentiated CAV status [31]. The presence of CAV was correlated with higher ECV and T2 and lower systolic/diastolic strain rates, supporting a tight coupling between diffuse structural changes and early functional impairment. The combination of all quantitative parameters achieved an AUC of 0.85 (95% CI 0.73–0.97), for detecting mild-moderate CAV and lead to a correct classification in 80% of patients as CAV-positive or CAV-negative [31]. These findings demonstrate that such multiparametric CMR has clear potential as a gatekeeper and adjunct to invasive angiography in HTx surveillance, particularly for detecting mild-moderate CAV that is angiographically subtle but clinically relevant.

### Quantitative approach by nuclear medicine procedures

Historically, nuclear imaging has played a central adjunctive role in the evaluation of the transplanted heart, primarily for the detection and risk stratification of CAV. Several studies have demonstrated that gated single-photon emission computed tomography (SPECT) is a well-established tool for myocardial evaluation in different patient populations, including individuals with suspected or known coronary artery disease and heart failure [32, 33]. In the transplant setting, gated SPECT has been used to assess regional perfusion defects, ventricular function, and to support risk stratification for cardiac allograft vasculopathy [34, 35]. Thus, this diagnostic method could play a significant role in the evaluation of HTx patients, with normal scans associated with a very low risk of hard cardiac events as cardiac death or re-transplantation and the potential to defer invasive angiography [34]. Moreover, even in the absence of overt ischemia, inhomogeneous stress perfusion patterns on myocardial SPECT were able to predict future allograft dysfunction over long-term follow-up, suggesting sensitivity to early microvascular or diffuse disease [35]. In parallel, cardiac PET was explored as a more quantitative research tool: early PET studies using tracers such as ^13^N-ammonia documented impaired myocardial flow reserve (MFR) in transplant recipients with angiographically normal coronaries and later demonstrated an inverse correlation between PET-derived perfusion reserve and IVUS measures of intimal thickening, highlighting microvascular involvement as an early manifestation of CAV [36]. Taken together, this “first wave” of nuclear cardiology established myocardial perfusion imaging, initially with SPECT and subsequently with PET, as a valuable non-invasive approach to screen for CAV, complement invasive angiography, and provide prognostic information in heart transplant recipients, even though it remained largely qualitative and centered on perfusion rather than fully quantitative flow metrics. Recent ISHLT guidelines for the care of HT recipients suggest that PET perfusion imaging and MBF quantification may be used for noninvasive detection of CAV and to provide prognostic information [10]. Advanced PET perfusion imaging with quantification of MBF and MFR offers robust assessment of microvascular dysfunction [37] and early manifestations of CAV that precedes angiographically visible stenosis [38]. Multiple studies showed that MBF quantification by PET in heart-transplant recipients has diagnostic and prognostic value for CAV identification and predicting outcomes such as graft loss or major adverse events [38–42]. Interesting, different studies showed that both hyperemic blood flow and MFR have been tested and specific cut-offs of MFR resulted extremely useful to identify CAV [38, 41, 42]. In a cohort of 181 recipients, Clerkin et al. [39] showed that a MFR ≤ 2.0 by ^13^N-ammonia PET identified patients at higher risk of death or re-transplantation, even without involvement of epicardial coronaries, establishing microvascular CAV as a powerful prognostic phenotype [39]. The diagnostic and prognostic value of ^82^Rb PET was recently tested and validated [41] developing a multiparametric non-invasive PET score including myocardial perfusion, absolute MBF quantification and allograft function to better define patient risk profile. In particular, using a quantification with specific MBF cut-off values in a per-vessels analysis, it was possible to identify the severity of CAV (mild, moderate and severe) [41]. In this study, the authors also underlined, the high NPV to rule out significant CAV by using ^82^Rb PET/CT imaging with regadenoson [41], as also demonstrated in published data [40]. Moreover, Prasad et al. [42] analyzed 344 transplant recipients and found that a ^13^N-ammonia PET-based CAV grade 2/3 (from MBF/MFR metrics) was associated with a 3 times higher risk of death or re-transplantation, while a negative PET had a high NPV for development of moderate-severe CAV on ICA over follow-up intervals up to four years [42]. These data underscore that PET-derived MBF/MFR metrics are independently prognostic and may allow extension of intervals between invasive coronary screenings in low-risk patients. Consequently, integration of these metrics into routine surveillance protocols may offer the potential to personalize follow-up intervals, guide immunosuppressive/anti-atherosclerotic therapies, and ultimately improve graft longevity and patient survival. Although PET-derived MBF quantification represents a highly promising tool for surveillance after HT, important logistical and economic barriers currently limit its widespread implementation in routine clinical practice. In particular, the use of ^13^N-ammonia is restricted by the requirement for an on-site cyclotron due to its short half-life, whereas ^82^Rb-chloride, although more operationally flexible, remains associated with substantial generator-related costs. In addition, quantitative PET perfusion imaging is presently concentrated in highly specialized tertiary referral centers with advanced nuclear cardiology infrastructure, which may limit the broader translatability of currently available evidence. Current real-world availability of quantitative PET myocardial perfusion imaging remains heterogeneous across transplant centers in Europe and North America. In Europe, quantitative PET perfusion imaging is still largely confined to academic or high-volume tertiary centers with dedicated nuclear cardiology expertise and access to advanced PET infrastructure, including on-site cyclotrons for tracers such as ^13^N-ammonia or specialized ^82^Rb generators. In North America, adoption appears somewhat broader, particularly in large academic transplant programs and tertiary referral centers in the United States and Canada, where generator-based ^82^Rb PET has facilitated clinical implementation. Nevertheless, availability remains incomplete. A recent analysis of Medicare-approved U.S. heart transplant centers demonstrated that only 55% had access to cardiac PET imaging, with marked geographic disparities and concentration in large academic centers [43].

## Latest findings in hybrid and molecular imaging

**Fig. 1:**
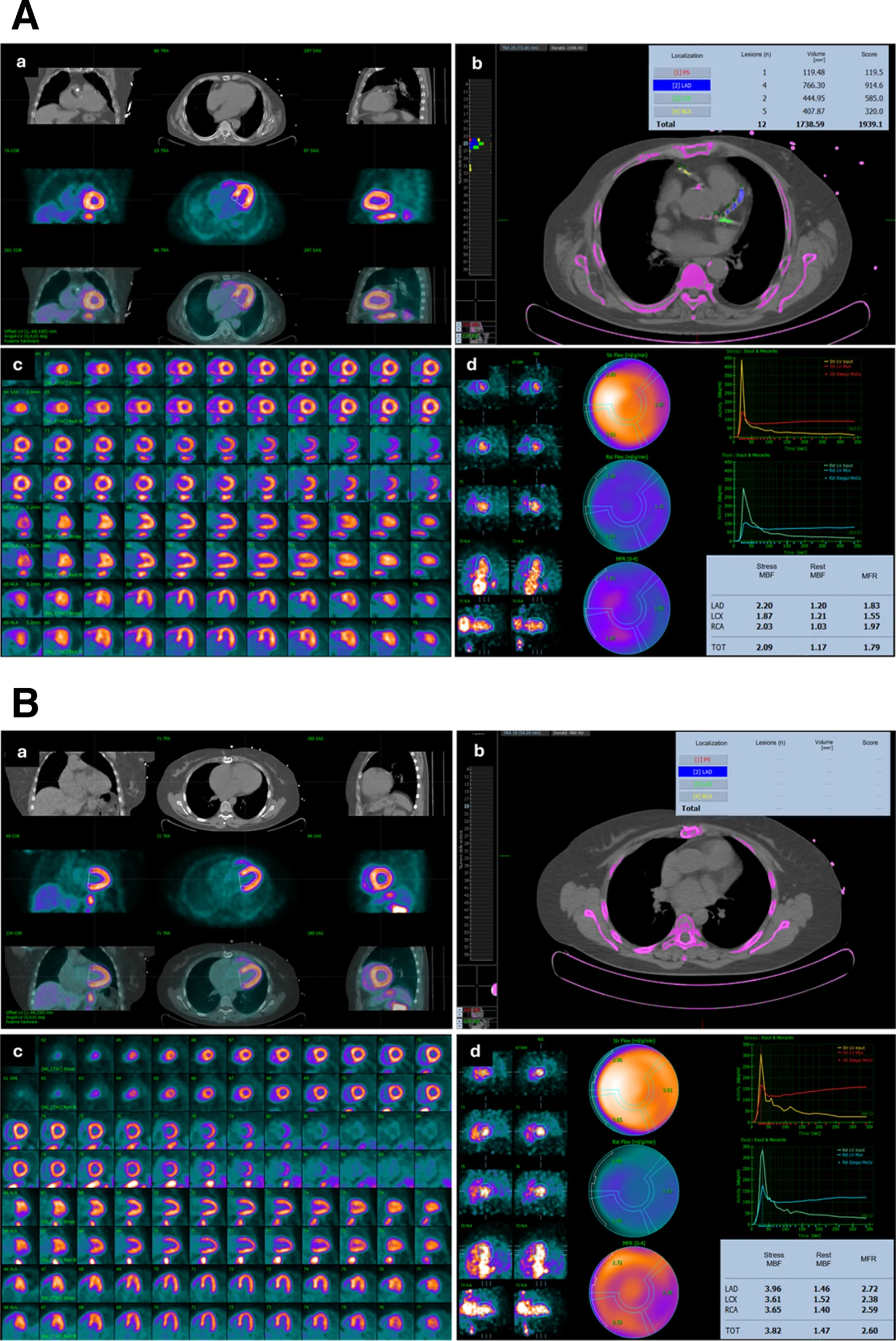
Examples of HTx patients undergoing ^82^Rb PET/CT imaging. **1 A** Patient with CAV showing normal perfusion, high calcium score and reduced quantitative MBF and MFR. **1B** Patient without CAV showing normal perfusion, no calcium score and normal quantitative MBF and MFR. **a**: transaxial CT, PET, and fusion imaging; **b**: perfusion by stress and rest perfusion imaging; **c**: calcium score quantification by CT images; **d**: quantitative MBF and MFR by blood flow quantification

A further step in this evolution towards predictive imaging is the use of hybrid imaging and novel PET tracers that integrate functional, structural, and molecular information within a single examination. Beyond perfusion-only PET, recent literature has highlighted the value of hybrid PET/CT for comprehensive assessment of HTx patients [44, 45]. Clerkin et al. [44] studied 430 adult recipients undergoing ^13^N-ammonia PET/CT and showed that visually estimated coronary artery calcium (CAC) derived from the low-dose CT of the perfusion study adds important anatomic risk information on top of physiologic PET assessment: patients with CAC 10–99 and ≥ 100 had a higher risk of death or re-transplantation versus patients with CAC 0, and this association remained significant after adjustment for conventional clinical and transplant predictors. In parallel, Sayed et al. [18] performed a systematic review and metanalysis of 44 studies (3,049 patients), demonstrating that coronary CTA has very high sensitivity and specificity for angiographic CAV, whereas PET-derived parameters such as MFR, stress MBF and PET-based CAV scores are strongly prognostic for major adverse events, formalizing the complementary strengths of anatomic CT and functional PET-derived parameters in the same surveillance framework. The findings of the recent paper by Assante et al. [45] illustrate the power of hybrid ^82^Rb PET/CT to characterize the transplanted coronary circulation at multiple levels in a single examination. They confirm that physiological vascular function, quantified by hyperemic MBF and especially MFR, adds substantial prognostic information. The presence of reduced MFR also in patients without CAV was associated with higher event rate, showing that microvascular dysfunction alone identifies a high-risk phenotype that would be potentially missed by IVUS. In addition, the CT-derived CAC score could represent an additional marker of chronic atherosclerotic and vasculopathy burden of the graft. Moreover, although epicardial adipose tissue (EAT) density did not independently predict events, its inverse association with MFR supports a pathophysiologic link between a pro-inflammatory perivascular context and impaired microcirculatory function [45]. These results reinforce the concept that the integration of macrovascular anatomy (CAV, CAC), microvascular function (MFR/MBF) and perivascular biology (EAT) within a single PET/CT session represent a valuable tool to refine risk stratification, personalize follow-up intervals, and target more aggressive cardioprotective or immunomodulatory strategies to patients with both structural and functional evidence of graft vasculopathy. In Fig. 1 is depicted ^82^Rb PET/CT imaging with simultaneous evaluation of perfusion, coronary vascular function, and plaque burden in an HTx patient with (Fig. 1A) CAV and without (Fig. 1B) CAV. Only few studies have tested the role of FDG-PET in the diagnosis of transplant rejection [46, 47]. Radionuclide imaging by SPECT and FDG-PET represent a non–invasive imaging modality used to detect myocardial viability and high metabolic activity in myocardial inflammatory conditions as sarcoidosis and myocarditis [48]. Given that myocardial rejection represents an inflammatory process, it is biologically plausible that ^18^F-FDG PET/CT can detect signs of myocardial rejection. Single-center retrospective studies have demonstrated that FDG uptake in the left ventricle was significantly elevated in the cardiac allograph rejection [46, 47]. The presence of persistent inflammation on FDG PET in some patients following treatment for rejection was indicated as possible parameter to help guide the decision to wean steroids treatment [46]. ^18^F-FDG PET/CT may represent a promising non–invasive diagnostic adjunct for detecting acute rejection following heart transplantation. Cardiac hybrid PET/MR combines high-resolution soft-tissue characterization (cine function, LGE, T1/T2/ECV mapping) with fully quantitative perfusion and metabolic/inflammatory PET, enabling simultaneous assessment of ventricular function, diffuse fibrosis, edema, perfusion, and inflammatory activity. Recent reviews on cardiac PET/MR emphasize that this hybrid approach improves diagnostic confidence and disease characterization in complex inflammatory and ischemic cardiomyopathies compared with stand-alone ^18^F-FDG PET or CMR [49]. Although formal heart-transplant cohorts are lacking in literature, these data support the concept that PET/MR could provide an integrated one-step assessment of rejection (inflammation/edema), diffuse interstitial remodeling (T1/ECV) and microvascular CAV (quantitative perfusion) in a single session. Parallel to hybrid hardware developments, novel PET tracers targeting fibroblast activation, immune cells and extracellular matrix remodeling are emerging as candidate biomarkers for early graft pathology [50–52]. Among these, tracers directed at fibroblast activation protein (FAP), such as ^68^Ga-FAPI, have generated intense interest [50]. FAPI PET has demonstrated myocardial uptake in inflammatory and fibrotic conditions such as myocarditis, systemic sclerosis and hypertrophic or dilated cardiomyopathy, often correlating with MRI evidence of fibrosis or impaired ventricular function [51]. These recent studies suggest that FAPI imaging may detect active fibro-inflammatory remodeling before, or in addition to, fixed scar on CMR, raising the possibility that in heart transplant recipients it could identify early fibrotic and immune-mediated injury that precedes overt graft dysfunction or angiographically visible CAV. In addition, immune-activation tracers and other non-FDG agents are being developed to improve the specificity of inflammatory imaging. A comprehensive 2024 review on novel PET radiotracers for myocardial inflammation highlights agents targeting somatostatin receptors, chemokine receptors such as CXCR4, integrins and translocator protein (TSPO), many of which have already shown promising results in sarcoidosis, myocarditis, and post-infarct inflammation [52]. Early feasibility protocols are beginning to include acute cellular cardiac allograft rejection as one of the target conditions for immune-targeted tracers (CXCR4-directed 68Ga-pentixafor), leading the way to non-invasive molecular imaging of alloimmune activation in the graft. While most of these data still derives from non-transplant populations, the underlying biology is shared with rejection and CAV, supporting the translational relevance of these tools [52].

## Artificial intelligence, radiomics and digital pathology

Artificial intelligence (AI) and radiomics are increasingly being explored as tools to move different fields of imaging from descriptive to predictive surveillance. Recent scoping reviews on AI and big data in heart transplantation highlight that machine-learning (ML) models can outperform traditional statistical scores in predicting graft failure, mortality, and other adverse outcomes, while also supporting decision-making along the entire transplant “supply chain”, from donor-recipient matching to post-transplant follow-up [53]. Table 3 summarizes the main findings of the most recent papers about the use of AI and ML in HTx patients. Registry-based studies have shown that survival ML models can match or outperform conventional risk scores for 1-year mortality after heart transplantation: ensemble ML in United Network for Organ Sharing (UNOS) data improved discrimination compared with traditional models [54], while single-center and national registry analyses from China and Sweden confirmed that ML models (random forests, gradient boosting, self-explaining neural networks) provide at least comparable AUC (0.63–0.70) with better calibration and allow identification of key drivers such as donor age, ischemic time and renal function [55, 56]. More recently, Liou et al. [57] applied multiple survival ML methods in the contemporary allocation era and showed that several algorithms achieve reasonable 1-year prognostic performance, but also that continual model updating is needed as practice evolves. In the specific context of rejection, several complementary AI approaches are emerging. At the tissue level, Lipková et al. [58] developed the CRANE system, a deep-learning framework trained on > 5,000 digitized EMB whole-slide images from three countries; the model simultaneously detected, subtyped, and graded rejection with AUC of 0.96 (95% CI: 0.943–0.980) and performance non-inferior to expert pathologists, while substantially reducing inter-observer variability. Deep-learning (DL) models trained on digitized EMB slides can now grade acute cellular rejection with performance comparable to expert pathologists, while reducing inter-observer variability: for example, an automated computational pipeline in a multicenter cohort showed that ML-derived rejection grades had good agreement with conventional ISHLT grading and were independently associated with clinical outcomes [59]. More recently, a self-supervised multiple-instance learning framework applied to routine biopsy slides demonstrated that AI could detect subtle morphologic patterns of rejection even in relatively small datasets, with AUC values in the 0.80–0.90 range for moderate-severe rejection [60]. Parallel advances in digital pathology suggest that computational pipelines will increasingly be integrated into transplant pathology workflows, standardizing EMB interpretation and enabling large-scale, image-based risk models [61]. Beyond tissue, AI-enabled non-invasive signals are opening new avenues for rejection prediction and surveillance. A deep-learning electrocardiogram (AI-ECG) model in HTx recipients achieved an AUC of 0.84 (95% CI: 0.78–0.90) for detecting moderate to severe acute cellular rejection, with excellent NPV, raising the possibility of frequent, low-cost remote screening to triage patients for EMB or advanced imaging [62]. In parallel, ML applied to circulating biomarkers is also emerging: a recent study using high-dimensional extracellular-vesicle profiles combined with AI achieved promising discrimination of rejection versus quiescent graft status, suggesting that multimodal models integrating imaging, digital pathology, ECG, and blood-based omics may ultimately provide the most powerful prediction of rejection risk [63]. Radiomics on the other hand, intended as the automated extraction of large numbers of quantitative texture and features from images, extends AI into the domain of imaging phenotypes. While transplant-specific radiomics studies are only beginning, there is rapidly growing evidence from related cardiac populations that radiomic signatures from CMR and PET capture microstructural and functional abnormalities invisible to the human eye [64, 65]. Texture analysis of T1 and T2 maps, for example, has shown to differentiate acute vs. chronic myocarditis and to provide incremental diagnostic information over mean mapping values alone [64]. Cine-CMR radiomics models have demonstrated prognostic value in dilated cardiomyopathy and other cardiomyopathies, adding independent information on arrhythmic or heart-failure risk beyond conventional volumetric and LVEF metrics [65]. These developments are directly relevant to transplantation, where diffuse fibrosis, microvascular CAV and low-grade rejection may all manifest as subtle, spatially complex texture patterns rather than overt LGE or mapping abnormalities. Moayedi et al. [66] applied supervised ML to quantitative intravascular ultrasound (IVUS) metrics in heart-transplant recipients and identified distinct CAV progression trajectories with differing event risks; their proof-of-concept model improved prediction of moderate-severe CAV and adverse outcomes compared with conventional IVUS thresholds alone, illustrating how ML can exploit serial vessel-level data to detect “hidden” progression. Although dedicated transplant imaging-radiomics cohorts are still limited, these examples illustrate how AI can exploit serial multimodal data to detect “hidden progression” and support dynamic risk reclassification over the graft’s lifetime. Despite this enthusiasm, substantial challenges in validation and clinical implementation remain. Reviews of AI in heart transplantation emphasize that most published models are single-center, retrospective and trained on relatively small, imbalanced datasets, raising concerns about overfitting, lack of generalizability and dataset shift when models are deployed in new populations or imaging protocols [53]. Annotation quality is another critical issue: EMB-based labels are subject to sampling error and inter-observer variability, which can propagate into AI models as noisy ground truth. Digital pathology and consensus pipelines may mitigate this, but they also introduce higher technical complexity and infrastructure requirements [61]. For radiomics, standardization of acquisition, reconstruction, segmentation, and feature-extraction is essential, as variations can dramatically alter feature distributions; recent work on repeatability and harmonization is encouraging but not yet fully translated into transplant-specific guidelines [67]. Regulatory and ethical considerations, including data privacy, algorithm transparency, bias across demographic subgroups, and the need for prospective, multi-centric, possibly randomized validation studies, are increasingly recognized as key hurdles before AI tools can be embedded in guideline-driven care pathways [53]. In summary, AI and radiomics in heart transplantation are moving rapidly from concept to early clinical proof-of-principle: multimodal AI models integrating imaging, clinical data, ECG, digital pathology and circulating biomarkers are beginning to predict rejection and CAV risk with higher accuracy than conventional approaches; radiomic signatures from CMR and PET show potential to capture diffuse, subclinical graft pathology and ML applied to serial follow-up can uncover hidden trajectories of progression. At the same time, rigorous technical standardization, external validation, and prospective outcome studies are urgently needed before these tools can be safely translated into routine surveillance protocols.

**Table 3:** Latest evidence about the use of AI and ML in heart transplanted patients

First author (Year)	Patient population	AI / ML Approach	Key findings
Ayers B. (2021) [55]	UNOS registry, adult isolated OHT	Ensemble ML on tabular clinical data	ML achieved higher discrimination and calibrated risk estimates, suggesting utility for candidate selection and counselling.
Zhou Y. (2021) [56]	Single-center Chinese cohort of orthotopic HTx recipients	Multiple ML algorithms (random forest, gradient boosting)	Best ML model reaching good AUC and identifying key predictors, illustrating feasibility in non-registry settings.
Lisboa P.J.G. (2022) [57]	National HT registry (Sweden)	Explainable AI (probabilistic neural network + LASSO)	AI model for long-term survival after HTx improved risk prediction vs. conventional models identifying key features driving mortality risk.
Liou L. (2025) [58]	Contemporary UNOS-linked cohort of adult HTx recipients (pre- vs. post-2018 allocation policy)	Survival ML (Random Survival Forests, Cox Gradient Boost)	Survival ML methods for 1-year mortality after HTx achieved reasonable performance.
Lipková J. (2022) [59]	5,056 endomyocardial biopsy whole-slide images from HTx recipients (multicenter, 3 countries)	Deep learning (CRANE) on digital EMB slides	Automated grading, subtyping and detection of cardiac allograft rejection reducing inter-observer variability and reading time.
Pevster (2021) [60]	2,472 EMB from 546 HTx recipients at 3 US centers	Computer-Assisted Cardiac Histologic Evaluation (CACHE)-Grader on digital EMB slides	The CACHE-Grader achieved 84.5% agreement, AUC 0.92 for high- vs. low-grade rejection, and agreement for 4-grade ISHLT classification, non-inferior to expert pathologists.
Seraphin T.P. (2023) [61]	Routine EMB whole-slide images in HTx recipients	Self-supervised deep learning, multiple-instance learning	DL can predict ISHLT rejection grade from routine WSIs with high AUC (0.80–0.90) even in smaller datasets; feasibility of data-efficient AI pathology for rejection.
Adedinsewo D. (2023) [63]	Adult HTx recipients with serial ECGs and paired biopsies	Deep-learning ECG (AI-ECG)	Developed AI-ECG model to detect moderate-severe acute cellular rejection
Burrello J. (2025) [64]	HTx recipients; plasma extracellular vesicle (EV) profiles	Machine learning on multi-omics EV signatures	ML-assisted EV profiling distinguished cellular rejection from quiescent graft
Moayedi Y. (2024) [67]	Adult HTx recipients with serial intravascular ultrasound (IVUS) and clinical follow-up	Supervised ML on quantitative IVUS features	ML model integrating IVUS metrics improved prediction of moderate-severe CAV and adverse outcomes vs. conventional cut-offs

## Clinical impact and future directions

The integration of advanced imaging biomarkers into post-transplant care offers significant promise for both patient outcomes and health-system efficiency. For example, a recent Australian randomized study in the first year after heart transplantation demonstrated that replacing routine invasive biopsy surveillance for rejection with a protocol based on CMR was cost-effective reducing biopsy volume by 94%, lowering costs by around AUD $32,900 and gained 0.588 quality-adjusted life-years (QALYs) without increasing risk of rejection or hospitalization [68]. More broadly, reviews of the cost-effectiveness of CMR across cardiac populations indicate that imaging-based surveillance is increasingly seen as a viable strategy for optimizing resource allocation while improving diagnostic accuracy [69]. From a health-systems perspective, the implication is that moving toward non-invasive quantitative imaging can reduce procedural risk, shorten hospital stays, and enable more tailored follow-up intervals, lightening resource capacity while potentially improving long-term graft survival at the same time. This shift also aligns with the broader trend toward value-based care and precision medicine. Moreover, because many of these newer biomarkers (tissue mapping, perfusion quantification) are reproducible and can be standardized across centers, there is clear potential for translation into practice guidelines. The current evidence on imaging in heart-transplant patients arises the need to incorporate non-invasive imaging into guideline-driven surveillance protocols. It should be considered that chronic kidney disease (CKD) is one of the main complications of HT patients (prevalence ranging between 10% and 80%) [70] representing an important determinant in the selection of imaging strategies for CAV surveillance for contrast-dependent modalities such as ICA and CCTA and contrast-enhanced CMR. In this context functional imaging approaches, including PET myocardial perfusion imaging and stress echocardiography, may offer practical advantages in selected patients with advanced CKD. Looking forward, opportunities for guideline integration include establishing threshold values for novel imaging quantitative biomarkers that should be validated in multi-centric studies and hopefully demonstrating that imaging-driven changes in therapy (immunosuppression adjustment, anti-atherosclerotic strategies) may lead to improved outcomes. Health-economics analysis will be crucial to demonstrate that upfront investment in advanced imaging may turn into reduced graft loss, fewer complications, and optimized follow-up strategies. In sum, the era ahead is likely to see imaging move from a descriptive adjunct to a central, guideline-embedded pillar of post-transplant management, supporting personalized surveillance, risk stratification, and resource-efficient care.

## Conclusions

Imaging in the setting of heart transplantation is undergoing a fundamental transformation from a descriptive to an integrated, predictive cornerstone of patient managementThe integration of multimodal and quantitative imaging approaches is now emerging as the future standard: parametric CMR mapping, PET perfusion, CT-derived functional metrics, and hybrid PET/MR molecular imaging are progressively moving the field beyond structural snapshots toward dynamic, mechanistic phenotyping. These modalities have demonstrated enhanced sensitivity for early graft changes and hold prognostic value for long-term graft survival and major adverse outcomes. Advanced imaging signatures may help to better define individual risk profiles for personalized long-term care of heart-transplant recipients. As evidence grows and standardization improves, these imaging advances are poised to be incorporated into practice guidelines, reshaping follow-up algorithms and shifting the paradigm from reactive detection of injury to proactive preservation of graft health. It has been specified that while imaging modalities have demonstrated promising diagnostic and prognostic performance, these findings should be interpreted with caution, as most published data originates from highly selected cohorts in specialized tertiary centers, which may limit their generalizability to routine clinical practice in unselected heart transplant populations.

## Data Availability

The datasets generated and analyzed during the current study are available from the corresponding author on reasonable request.
